# Hiatal hernia prevalence and natural history on non-contrast CT in the Multi-Ethnic Study of Atherosclerosis (MESA)

**DOI:** 10.1136/bmjgast-2020-000565

**Published:** 2021-03-17

**Authors:** Jinhye Kim, Grant T Hiura, Elizabeth C Oelsner, Xiaorui Yin, R Graham Barr, Benjamin M Smith, Martin R Prince

**Affiliations:** 1 Department of Radiology, Weill Cornell Medicine, New York, NY, USA; 2 Department of Medicine, Columbia University Irving Medical Center, New York, NY, USA; 3 Department of Epidemiology, Mailman School of Pubilc Health, Columbia University, New York, NY, USA; 4 Department of Medicine, McGill University, Montreal, QC, Canada; 5 Department of Radiology, Columbia University Irving Medical Center, New York, NY, USA

**Keywords:** gastric diseases, gastroesophageal reflux disease, hiatal hernia, oesophagus-gastric junction, radiology

## Abstract

**Objective:**

To determine the prevalence, risk factors and natural history of hiatal hernia (HH) on CT in the general population.

**Materials and methods:**

The Multi-Ethnic Study of Atherosclerosis (MESA) acquired full-lung CT on 3200 subjects, aged 53–94 years. Three blinded observers independently determined presence/absence and type (I–IV) of HH. Associations between HH and participant characteristics were assessed via unadjusted and multivariable-adjusted relative risk regression. HH natural history was assessed compared with prior MESA CT.

**Results:**

Excellent interobserver agreement was found for presence (κ=0.86) and type of HH (κ=0.97). Among 316 HH identified (prevalence=9.9%), 223 (71%) were type I and 93 (29%) were type III. HH prevalence increased with age, from 2.4% in 6th decade to 16.6% in 9th decade (unadjusted prevalence ratio (PR)=1.1 (95% CI 1.04 to 1.1)). HH prevalence was greater in women (12.7%) than men (7.0%) (unadjusted PR=1.8 (95% CI 1.5 to 2.3)) and associated with proton pump inhibitor use (p<0.001). In 75 participants with HH with 10-year follow-up, median HH area increased from 9.9 cm^2^ to 17.9 cm^2^ (p=0.02) with a higher mean body mass index (BMI) in subjects with increasing HH size compared with HH decreasing in size: mean BMI=30.2±6.2 vs 26.8±7.2 (p=0.02).

**Conclusion:**

HH on non-contrast CT is prevalent in the general population, increasing with age, female gender and BMI. Its association with proton pump inhibitor use confirms a role in gastro-oesophageal reflux disease and HH progression is associated with increased BMI.

**Trial registration number:**

NCT00005487.

**Key messages**

**What is already known about this subject?**

- Hiatal hernia (HH) prevalence and risk factors have been studied extensively in the population of patients undergoing endoscopy, but the prevalence of HH in the general population is unknown.

**What are the new findings?**

- Non-contrast CT on 3200 Multi-Ethnic Study of Atherosclerosis subjects followed up over 10 years shows HH prevalence in the general population (aged 53–94 years) increases with ageing from 2.4% in the sixth decade of life to 7.0%, 14.0% and 16.6% in seventh, eighth and ninth decades, respectively and is more common in women (PR=1.8) and in those with obesity (PR=1.1).
- HH on CT scans is associated with proton pump inhibitor use and is more likely to progress in subjects with high body mass index (BMI).

**How might it impact on clinical practice in the foreseeable future?**

- Finding HH on chest CT raises the likelihood of gastro-oesophageal reflux disease and patients with high BMI have an association with HH progression.

## Introduction

Hiatal hernia (HH) is a common incidental finding on radiological and endoscopic studies.^1–5^ Both the anatomical (HH) and the physiological (lower oesophageal sphincter) features of the gastro-oesophageal (GE) junction are considered to be important in the pathogenesis of gastro-oesophageal reflux disease (GERD).^3 6^ GERD may also contribute to HH development when acid exposure causes oesophageal mucosal injury, which may lead to oesophageal shortening, thus ‘pulling’ the GE junction into the chest.^7^

Prior literature has identified potential risk factors for HH, including older age, pregnancy and obesity.^5 8–15^ However, these studies have been limited to specific symptomatic patient populations (eg, undergoing endoscopy) or with pulmonary conditions exacerbated by reflux, including idiopathic pulmonary fibrosis, chronic obstructive pulmonary disease and asthma.^3 6 8 16–21^ Studies establishing the prevalence and correlates of HH in a general, population-based sample are lacking.

In this study, we used CT scans from the Multi-Ethnic Study of Atherosclerosis (MESA) to assess the general population-based prevalence, risk factors and natural history of HH over 10-year follow-up.

## Materials and methods

### Study population

**Figure 1:**
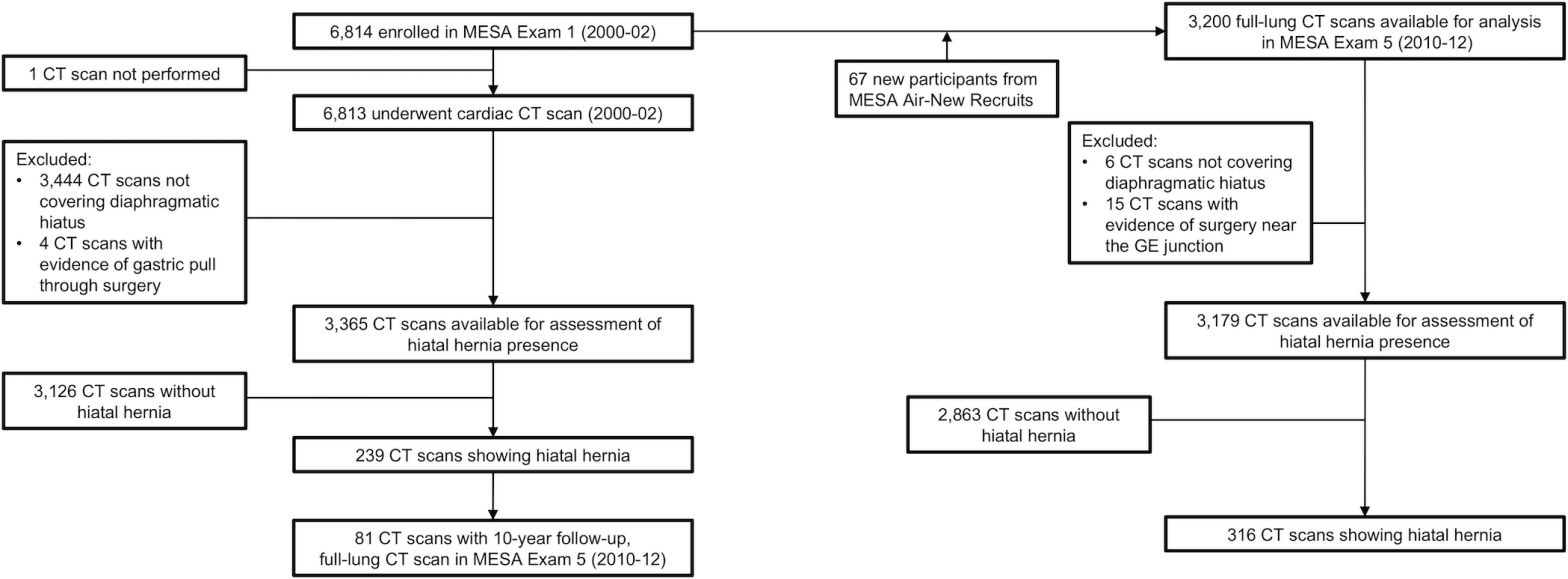
Subject recruitment flow chart. GE, gastro-oesophageal; MESA, Multi-Ethnic Study of Atherosclerosis.

MESA is a prospective multisite cohort study investigating the prevalence, correlates and progression of subclinical cardiovascular disease.^22^ In 2000–02, MESA recruited 6814 participants aged 45–84 years from six US communities. Exclusion criteria included clinical cardiovascular disease (physician diagnosis of heart attack, stroke, transient ischaemic attack, heart failure, angina, current atrial fibrillation, any cardiovascular procedure), weight over 136 kg, pregnancy or any impediment to long-term participation.^22^ At MESA Exam 1, 6813 participants underwent cardiac CT. During 2010–12, MESA Exam 5 acquired full-lung CT scans from 3200 participants, including 67 participants who were additionally recruited for the MESA Air Study in 2005–07 (figure 1).^23^

### CT scanning

MESA Exam 5 acquired full-lung CT scans at full inspiration. The centres used four models of 64-slice multidetector row CT scanners from two manufacturers (Siemens Medical Solutions, Erlangen, Germany and GE Healthcare, Waukesha, Wisconsin, USA). The protocol for scanning was based on Subpopulations and Intermediate Outcome Measures in COPD Study/MESA Lung CT protocol.^23^ Images were reconstructed at 0.625 mm slice thickness.^23^

MESA Exam 1 scans used cardiac-gated electron-beam CT scanners (Imatron C-150) or a prospectively electrocardiogram-triggered multidetector CT acquisition at 50% R-R interval acquiring a block of four axial 2.5 mm slices during each cardiac cycle sequentially (GE Lightspeed or Volume Zoom Siemens) using parameters reported previously.^22^

### Image analysis

**Figure 2:**
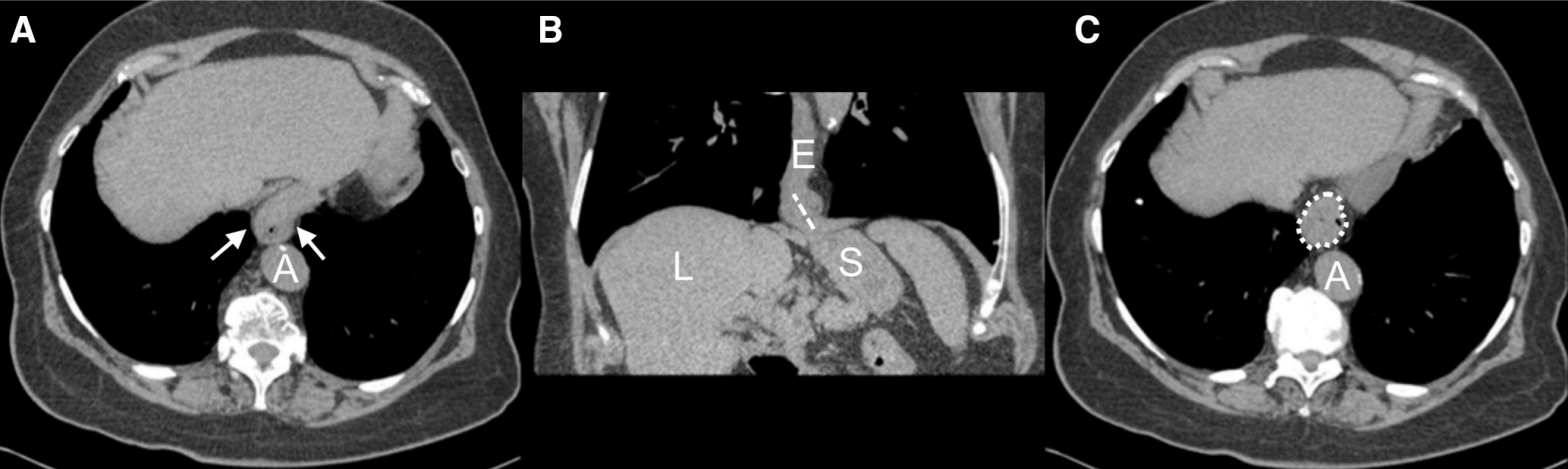
Non-contrast chest CT of a woman aged 84 years. (A) Axial image showing type I hiatal hernia (white arrows). (B) Coronal oblique reformation shows gastric folds extending 2.9 cm (dashed line) above the diaphragm. (C) Axial image shows maximum hiatal hernia cross-sectional area measurement (dotted line, 7.6 cm^2^). A, aorta; E, oesophagus; L, liver; S, stomach.

**Figure 3:**
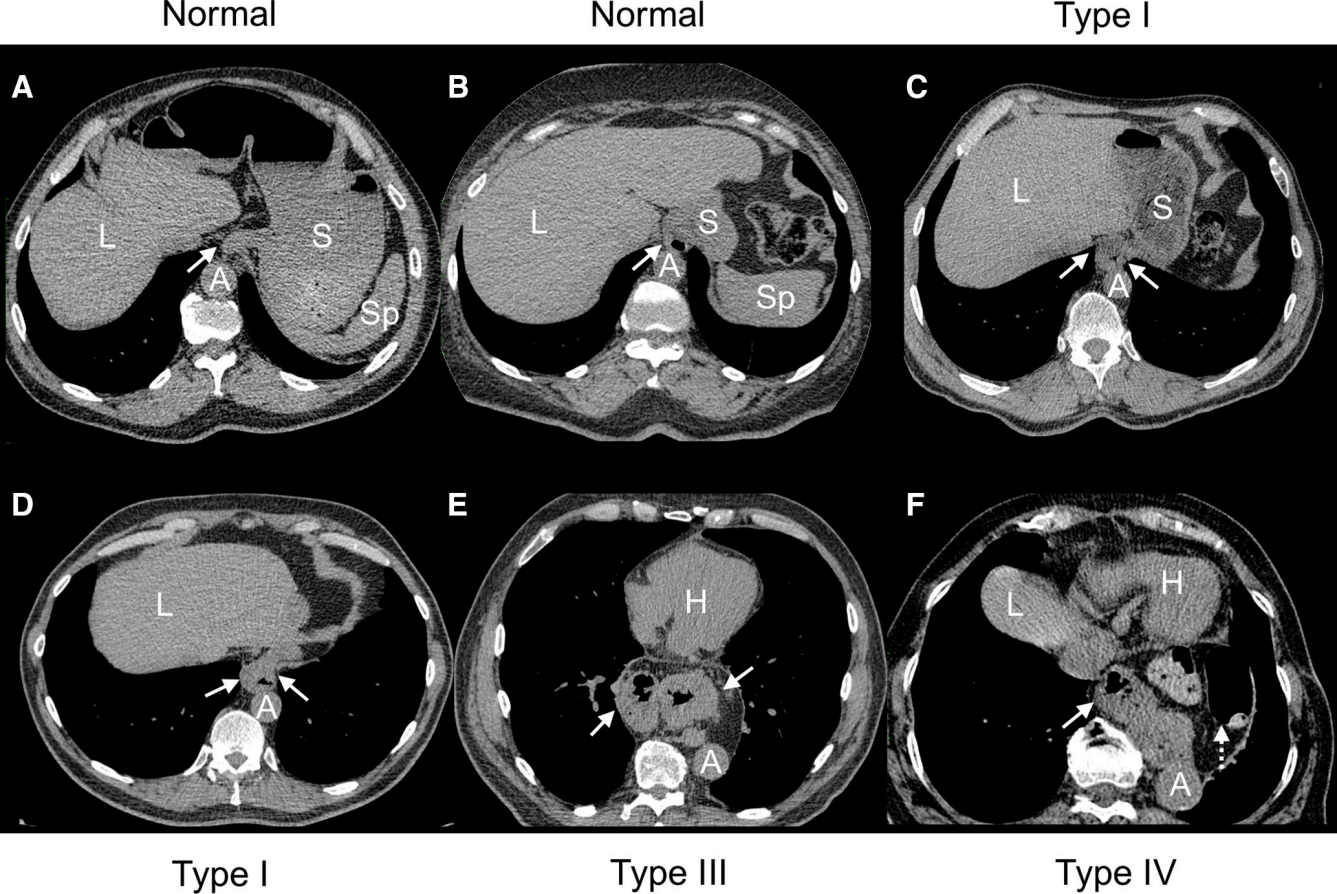
Examples of normal gastro-oesophageal (GE) junction and types I, III and IV hiatal hernia (HH) on non-contrast CT. (A) Non-contrast chest CT of a man aged 80 years with three reviewers reporting normal GE junction (arrow). (B) Non-contrast chest CT of a woman aged 59 years with type 1 HH (arrow) reported by one of three reviewers and considered normal by the other two reviewers. (C) Non-contrast chest CT of a man aged 85 years with two out of the three reviewers reporting type I HH (arrows). (D) Non-contrast chest CT of a man aged 64 years with all three reviewers reporting type I HH (arrows). (E) Non-contrast chest CT of a man aged 75 years with three reviewers reporting type III HH (arrows). (F) Non-contrast chest CT of a woman aged 84 years with three reviewers reporting type IV HH (solid arrow) with colon in the thorax (dashed arrow). A, aorta; H, heart; L, liver; S, stomach; Sp, spleen.

Since MESA Exam 5 full-lung CT scans had thinner scan slice thickness compared with MESA Exam 1 cardiac CT scans and more consistently covered down to the GE junction, our study mainly used Exam 5 data. MESA Exam 1 cardiac CT scans were also analysed and used as supplementary data. CT scans were analysed using Horos (https://horosproject.org/, open source medical image viewer). A subset of MESA Exam 1 cardiac CT scans (n=393) and MESA Exam 5 full-lung CT scans (n=1031) were reviewed independently by three observers (JK, XY, MRP) blinded to the participants’ information to calculate interobserver agreement. The remainder of the CT scans were reviewed by a single observer (JK). CT scans were evaluated for presence of HH, defined as gastric folds extending >2 cm above the diaphragm on axial images and/or reformations measured using electronic callipers (figure 2). Each HH identified was classified as type I–IV, as previously described,^20^ by the three independent observers (figure 3). Discrepancies among reviewers in evaluating for the presence of HH and final type of HH were resolved by majority opinion. The largest cross-sectional HH area (cm^2^) on axial images was measured using the closed polygon tool. Location of the hernia relative to aorta (left, right or midline) was noted. To ensure that only CT scans with an adequate coverage of the diaphragmatic hiatus were included, any CT scans for which any of the three observers noted inadequate coverage of the diaphragmatic hiatus were excluded from further analyses. Participants with HH at both MESA Exams 1 and 5 were additionally evaluated for changes in HH type and maximum hernia cross-sectional area.

### Data collection

Characteristics of MESA participants including demographics (eg, age, gender, race/ethnicity), anthropometry (eg, height, weight, body mass index (BMI), waist and hip circumference), smoking behaviours, alcohol use, comorbidities, medication use and parity were collected. Age was treated as both continuous and categorical by decade. Education was re-categorised into five groups (<high school, high school graduate, some college, college graduate, >bachelor’s degree). Central obesity was defined as waist-to-hip ratio >0.9 for males and >0.85 for females.^24^ As in prior studies, number of live births was categorised as 0, 1–2, 3–4 and >5.^25^

### Statistical analysis

Fleiss’ kappa was used to assess reproducibility of HH presence, type and determination of diaphragmatic coverage on Exam 1 cardiac CT scans. The intraclass correlation coefficient (ICC) was used to assess reproducibility of HH measurements (largest cross-sectional HH area and length of gastric folds above the diaphragm). Kappa and ICC values closer to 1 represent stronger reproducibility. The consensus types (ie, at least two raters agreed) and mean area measurements (ie, across all three raters) were used for all subsequent analyses.

Bivariate associations between predictor variables and HH presence were assessed using relative risk regression. Prevalence ratios were calculated using Poisson regression with robust error variance. Elastic net regression was used for model selection and variables above the optimal value of the criterion threshold were considered for multivariable analyses. For participants with HH at both Exams 1 and 5, per cent change of HH maximal cross-sectional area was calculated, as $\frac{Exam5area-Exam1area}{Exam1area}\times 100$.

Cox proportional hazards regression was used to model HH incidence from Exams 1 to 5 among participants free of HH at baseline. These models were adjusted for the potential confounders (age, sex, race/ethnicity, height and weight). The effect of intervertebral disc and vertebral body compression on the incidence of HH was measured by modelling the loss of height from Exam 1 to Exam 5, as a continuous variable, in a logistic regression model with incident HH at Exam 5 as the outcome. All analyses were performed in SAS V.9.4 (SAS Institute, Cary, North Carolina, USA). Full MESA study protocol can be accessed at https://clinicaltrials.gov. There is no overlap with other MESA publications, https://www.mesa-nhlbi.org/Publications.aspx.

### Patient and public involvement

Given the retrospective analysis of existing data, it was not appropriate or possible to involve patients or the public in the design, or conduct, or reporting, or dissemination plans of our research.

## Results

### Demographic data

The characteristics of study participants are summarised in table 1. In MESA Exam 5, participants (n=3200) completed full-lung CT scans. Twenty-one scans were excluded: 6 scans did not extend sufficiently inferiorly to completely image the diaphragmatic hiatus, and 15 scans had evidence of surgery (eg, surgical clips) near the GE junction (figure 1).

**Table 1:** Characteristics of study participants at MESA Exam 5 (n=3179)

Variable	Non-contrast chest CT scans (MESA Exam 5)			
	HH (n=316)	No HH (n=2863)	HH prevalence ratio* (95% CI)	P value
Age (years), mean±SD	73.9±8.2	68.8±9.2	1.05 (1.04 to 1.06)	<0.001*
<50, n (%)	0 (0%)	0 (0%)		
50–59	13/316 (4%)	522/2863 (18%)	Ref	Ref
60–69	79/316 (25%)	1048/2863 (37%)	2.88 (1.62 to 5.14)	<0.001*
70–79	143/316 (45%)	876/2863 (31%)	5.78 (3.31 to 10.1)	<0.001*
80–89	78/316 (25%)	392/2863 (14%)	6.83 (3.85 to 12.1)	<0.001*
>90	3/316 (1%)	25/2863 (1%)	4.41 (1.33 to 14.6)	0.02*
Gender				
Male	106/316 (34%)	1413/2863 (49%)	Ref	Ref
Female	210/316 (66%)	1450/2863 (51%)	1.81 (1.45 to 2.27)	<0.001*
Race/Ethnicity				<0.001*
White	149/316 (47%)	1084/2863 (38%)	Ref	Ref
African-Americans	81/316 (26%)	780/2863 (27%)	0.78 (0.60 to 1.01)	0.06
Hispanic	74/316 (23%)	597/2863 (21%)	0.91 (0.70 to 1.19)	0.49
Asian-Americans	12/316 (4%)	402/2863 (14%)	0.24 (0.13 to 0.43)	<0.001*
Height (cm)	162.2±9.7	165.8±9.9	0.97 (0.96 to 0.98)	<0.001*
Weight (lb)	174.3±34.7	172.4±39.1	1.00 (1.00 to 1.00)	0.37
BMI (kg/m^2^)	30.0±5.5	28.3±5.5	1.05 (1.03 to 1.06)	<0.001*
Circumference (cm)				
Waist	103±14.3	98.9±14.3	1.02 (1.01 to 1.02)	<0.001*
Hip	109.0±12.9	105.2±12.0	1.02 (1.01 to 1.03)	<0.001*
Waist-to-hip ratio	94.7±8.7	93.9±8.0	1.01 (1.00 to 1.02)	0.10
Central obesity†	278/316 (88%)	2380/2858 (83%)	1.42 (1.03 to 1.97)	0.03*
Body surface area	1.84±0.20	1.85±0.23	0.72 (0.47 to 1.09)	0.12
Education				0.09
<High school	51/316 (16%)	386/2859 (13%)	Ref	Ref
High school graduate	65/316 (21%)	497/2859 (17%)	0.99 (0.70 to 1.40)	0.96
Some college	96/316 (30%)	819/2859 (29%)	0.90 (0.65 to 1.24)	0.51
College graduate	46/316 (15%)	542/2859 (19%)	0.67 (0.46 to 0.98)	0.04*
>Bachelor’s degree	58/316 (18%)	615/2859 (21%)	0.74 (0.52 to 1.05)	0.10
Cigarette smoking				0.004*
Never smoker	151/315 (48%)	1297/2861 (45%)	Ref	Ref
Former smoker	147/315 (47%)	1276/2861 (45%)	0.99 (0.80 to 1.23)	0.93
Current smoker	17/315 (5%)	288/2861 (10%)	0.53 (0.33 to 0.87)	0.01*
Pack-years	24.4±26.5	22.2±25.8	1.00 (1.00 to 1.01)	0.10
Diabetes				0.81
No diabetes	188/315 (60%)	1666/2842 (59%)	Ref	Ref
Impaired fasting glucose	68/315 (21%)	601/2842 (21%)	1.00 (0.77 to 1.30)	0.99
Diabetes	59/315 (19%)	575/2842 (20%)	0.92 (0.69 to 1.21)	0.55
Self-reported symptoms				
Emphysema/COPD	6/314 (2%)	53/2849 (2%)	1.02 (0.48 to 2.20)	0.95
Asthma	15/315 (5%)	93/2856 (3%)	1.42 (0.88 to 2.30)	0.16
Bronchitis, past 2 weeks	4/315 (1%)	37/2850 (1%)	0.98 (0.38 to 2.50)	0.97
Medications				
Proton pump inhibitors	74/316 (23%)	344/2863 (12%)	2.02 (1.59 to 2.57)	<0.001*
H2 blockers	14/316 (4%)	84/2863 (3%)	1.46 (0.89 to 2.39)	0.14
Insulin or oral hypoglycaemics	48/315 (15%)	464/2846 (16%)	0.93 (0.69 to 1.25)	0.63
Ever been pregnant (women)	177/201 (88%)	1235/1425 (87%)	1.12 (0.75 to 1.67)	0.59
# of pregnancies	3.5±1.9	3.4±2.1	1.01 (0.96 to 1.07)	0.67
Age at first live birth	23±4.7	24±5.4	0.98 (0.96, 1.01)	0.14

*HH prevalence ratio=the prevalence of HH for the group defined by the variable in column 1.

†Central obesity is defined as a waist-to-hip ratio >0.90 for males and >0.85 for females.

BMI, body mass index; COPD, chronic obstructive pulmonary disease; HH, hiatal hernia; MESA, Multi-Ethnic Study of Atherosclerosis.

Of the 6813 study participants with MESA Exam 1 cardiac CT scans, 3444 (51%) had scans that did not cover the diaphragmatic hiatus, 26 of which showed stomach herniated up into the thorax. However, we decided a priori to only include scans that covered the diaphragmatic hiatus. Four participants who received gastric pull-through surgery were also excluded.

### Interobserver agreement

In Exam 5 full-lung CT scans, interobserver agreement was high for determining HH presence (κ=0.86 (95% CI 0.8 to 0.9) and HH type (κ=0.97 (95% CI 0.9 to 0.99)). Interobserver agreement was also high for the quantitative HH measures: for the length of gastric folds above the diaphragm, the ICC was 0.94, and for the maximum hernia cross-sectional area, the ICC was 0.99.

In Exam 1 cardiac CT scans, interobserver agreement was high for identifying cardiac CT scans with adequate coverage of the diaphragmatic hiatus (κ=0.88 (95% CI 0.8 to 0.9)), and determining the type of HH (κ=0.85 (95% CI 0.8 to 0.9)). For the maximum hernia cross-sectional area, the ICC was 0.99.

### Prevalence and characteristics of hiatal hernia

Among 3179 participants with Exam 5 full-lung CT scans, 316 HHs were identified (prevalence=9.9%), including 223 type I and 93 type III (figure 2). The median HH size in the axial plane (ie, maximum cross-sectional area) was 7.1 cm^2^ (IQR 5.6–16.0). The median length of gastric folds above the diaphragm of the type I HH was 2.4 cm (IQR 2.1–2.8) and 6.1 cm (IQR 5.0–7.5) for type III HH. The locations of HHs relative to aorta were midline for 307 (97%) with 9 (3%) on the left.

In bivariate analyses, HH prevalence increased with age, from 2.4% in the sixth decade of life to 7.0%, 14.0% and 16.6% in seventh, eighth and ninth decades, respectively (table 1). The prevalence of HH was 10.7% for participants 90 years of age or older. Participants with HH were significantly older than the participants without HH (p<0.001). HH presence was greater in women (12.7%) than in men (7.0%) (prevalence ratio (PR)=1.8 (95% CI 1.5 to 2.3)). HH prevalence varied by race/ethnicity, showing higher prevalence in non-Hispanic whites (12.1%), African-Americans (9.4%) and Hispanic/Latinos (11.0%) and lower prevalence in Asian-Americans (2.9%) (p<0.001). Other associations were found for markers related to obesity (BMI, waist circumference, hip circumference, central obesity), height, educational attainment, current smoking status and proton pump inhibitor use.

The top predictors from elastic net selection were age, gender, race/ethnicity and BMI. After adjusting for these variables, HH remained associated with proton pump inhibitor use (PR=1.6 (95% CI 1.2 to 2.0)), but was not significantly associated with cigarette smoking status (p=0.19), number of pregnancies (p=0.42) or number of live births (p=0.99) (table 2).

**Table 2:** Multivariable models for presence of hiatal hernias at MESA Exam 5

Variable	N	Prevalence ratio (95% CI)	P value
Waist-to-hip ratio	3171	1.01 (1.00 to 1.02)	0.12
Central obesity	3171	1.27 (0.91 to 1.76)	0.16
Cigarette smoking status	3176		0.19
Never smoker		Ref	Ref
Former smoker		0.91 (0.74 to 1.13)	0.39
Current smoker		0.68 (0.42 to 1.10)	0.11
Medications			
Proton pump inhibitor	3176	1.57 (1.24 to 2.00)	<0.001*
H2 blockers	3176	1.16 (0.70 to 1.93)	0.57
Bronchitis, last 2 weeks	3162	0.93 (0.36 to 2.42)	0.89
Women only			
# of pregnancies	1415	0.98 (0.92 to 1.03)	0.42
# of live births	1415		0.99
0		Ref	Ref
1–2		1.06 (0.56 to 1.98)	0.87
3–4		1.05 (0.55 to 2.00)	0.88
>5		1.11 (0.55 to 2.22)	0.77

Adjusted for age, gender, race/ethnicity and BMI.

BMI, body mass index; MESA, Multi-Ethnic Study of Atherosclerosis.

We also assessed the prevalence and characteristics of HH in Exam 1 cardiac CT scans. Among the 3365 participants with scans including the diaphragmatic hiatus, 239 HHs were identified (prevalence=7.1%), including 145 type I, 93 type III and 1 type IV HH (figure 3). The median maximal HH cross-sectional area was 9.0 cm^2^ in the axial plane. The locations of HH relative to aorta were mostly midline (97.0%) with more to the left (2.5%) than to the right (0.4%) of the aorta.

Bivariate analyses of HH presence in Exam 1 cardiac CT scans showed similar results to those in Exam 5 full-lung CT scans. However, in Exam 1, self-reported bronchitis (within past 2 weeks), H2 blocker use and current use of hormone replacement therapy were additionally associated with HH presence. In Exam 1, the top predictors from elastic net selection were the same as in Exam 5, and after adjustment for these variables, HH remained associated with proton pump inhibitor use (p*<*0.001) and waist-to-hip ratio (p=0.049). There were no significant associations between HH and cigarette smoking status (p=0.28), alcohol use (p=0.69), number of pregnancies (p=0.43) or number of live births (p=0.29).

### Incidence of hiatal hernia over 10-year follow-up

Among 1464 participants free of HH on baseline Exam 1 cardiac CT, the incidence rate of HH was 9 per 1000 person-years. In adjusted models, the risk of developing HH increased with weight (HR=1.01 (95% CI 1.01 to 1.02)), and was lower among Asian-Americans compared with non-Hispanic whites (HR=0.4 (95% CI 0.2 to 0.9)). For age, the incidence was always positive, consistent with increasing HH prevalence with ageing, but was less positive with increasing age (HR=0.3 (95% CI 0.3 to 0.4)). HH also occurred more frequently in patients with loss of height between Exams 1 and 5 (p=0.0005) (table 3).

**Table 3:** Association between amount of change in height from MESA Exam 1–5 and incidence of HH (n=1464)

Loss of height from Exam 1–5	Number without HH at Exam 1	Number developing HH between Exams 1 and 5	% with HH	P trend
All	1464	133	9.1	0.0005*
<1 cm	547	38	7.0	
1–2 cm	507	42	8.3	
2–3 cm	255	29	11.4	
3–4 cm	96	14	14.6	
4–5 cm	34	6	17.7	
>5 cm	25	4	16.0	

P trend was measured by modelling the ordinal predictor, loss of height from Exam 1–5, as a continuous variable in a logistic regression model, with incident HH at Exam 5 as the outcome.

HH, hiatal hernia; MESA, Multi-Ethnic Study of Atherosclerosis.

### Natural history of hiatal hernia

**Figure 4:**
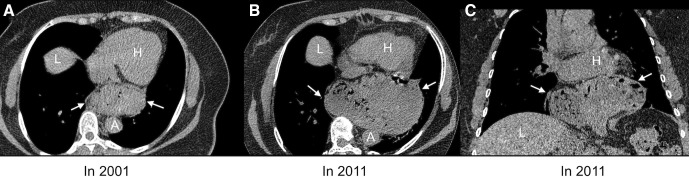
Non-contrast chest CT of a woman aged 70 years showing natural history of hiatal hernia (HH) over 10 years from Multi-Ethnic Study of Atherosclerosis (MESA) Exam 1 (2000–02) to MESA Exam 5 (2010–12). (A) Axial image of type III HH (arrows) at MESA Exam 1 (2001). (B) Increased size of type III HH (arrows) at MESA Exam 5 (2011). (C) Coronal reformation at MESA Exam 5 showing most of stomach herniated into thorax (arrows). A, aorta; H, heart; L, liver; S, stomach.

Eighty-one of the 239 subjects with HH at Exam 1 had 10-year follow-up CT scans, 6 of whom no longer had HH on follow-up imaging, including one participant whose HH was surgically repaired and 5 participants (6.3%) whose HH spontaneously resolved. Among 75 participants with HH at Exams 1 and 5, HH type was unchanged for 26 participants with type I HH and 34 participants with type III HH. Progression from type I to type III was observed in 12 participants. The median maximal cross-sectional area of HH increased from 9.9 cm^2^ to 17.9 cm^2^ (p=0.02, figure 4). For those subjects whose HH spontaneously resolved (n=5) or became >10% smaller in area (n=10), the mean weight loss from Exam 1 to Exam 5 was 6.9±6.5 kg compared with 2.6+7.5 kg for the other 65 subjects with HH at both exams (p=0.05).

For per cent change, the median was 36.4% (IQR 2.1–110.8). After excluding the one subject who had HH repaired, we categorised our 80 subjects with 10-year follow-up CT scans into three groups according to per cent change: ‘area increased by >10%’ (n=19), ‘area decreased by >10% or HH spontaneously resolved’ (n=15) and ‘area change was within 10%’ (n=46). The BMI of the subjects who showed decrease in HH area >10% or spontaneous resolution of HH (mean±SD: 26.8±7.2) was significantly lower compared with that of the subjects who showed >10% increase of HH area (mean+SD: 30.2+6.2) (p=0.02).

## Discussion

In spite of the clinical significance of HH—its role in GERD,^3 6^ its association with aspiration and lung disease,^16–21 26 27^ and the potential for reducing quality of life^28–30^—little is known about HH prevalence, risk factors and natural history in the general population. This is due, at least in part, to the fact that traditional measures of HH, such as endoscopy, manometry or barium swallow radiography, are rarely performed in asymptomatic subjects. This MESA study involving 3179 subjects free of cardiovascular disease shows non-contrast chest CT is highly reproducible for detecting and typing HH and confirms that HH on CT is common in the general population increasing in prevalence with age, female gender and BMI. High BMI is further associated with increasing HH size over a 10-year follow-up.

Our observation of an age-dependent relationship of HH detected by CT is consistent with a meta-analysis of endoscopy studies,^5^ which showed a similar significant association of HH prevalence with age above 50 years. This adds to the confidence in these CT results. This age dependence may reflect decreasing elasticity of the phreno-oesophageal ligament, which normally anchors the oesophagus to the diaphragm but progressively weakens with ageing, increasing HH risk. One aberration from this age dependence was a lower prevalence of HH among participants in their 90s, 10.7% (Exam 5), compared with their 80s, 22.7% (Exam 1). This finding suggests a survival bias and could relate to previously established associations between HH and aspiration.^16^

Our study also showed significant associations between BMI and HH prevalence, which is in agreement with findings from the prior literature.^5^ The significant difference in BMI between the groups of participants with progression of HH (ie, HH area increased >10% over 10 years) and improvement of HH (ie, HH area decreased >10% or HH spontaneously resolved) raises the possibility that obesity, which increases intra-abdominal pressure, may play a role in the development and progression of HH. Another mechanism that could contribute to observed associations of HH with both increasing age and female sex is osteoporosis, with loss of vertebral body and intervertebral disc space height reducing space available in the abdomen to accommodate intra-abdominal organs.^31^ Our data here showing incidence of HH over a 10-year interval correlating with loss of height over the same time interval supports this hypothesis. Observing HH to resolve over time is contrary to its pathophysiology and thus may reflect the threshold for detection being met on the initial exam but not subsequently, especially since these patients had weight loss between Exams 1 and 5 which might allow a sliding hiatal hernia to reduce in size.

Although our observation of higher HH prevalence females contrasts with the prior meta-analysis,^5^ in that endoscopy study only 38.8% of subjects were male indicating a bias towards undersampling males. Less symptomatic males (with lower likelihood of HH) refusing invasive endoscopy may explain those meta-analysis data which also had high heterogeneity (I^2^=90%). Also contrary to some prior studies,^32 33^ we did not identify significant associations between HH and parity (number of pregnancies, p=0.42; number of live births, p=0.99). This may relate to the advanced age of our population, since pregnancy-induced HH could resolve spontaneously over time.

HH on CT was less common in Asian-Americans compared with other race/ethnic groups consistent with GERD being uncommon in Asian countries compared with the western world.^34^ Kang and Ho^35^ showed that reflux oesophagitis and HH are more common in English dyspeptic patients compared with Singaporeans. Considering that obesity has been recognised as an important HH risk factor,^5^ one theory to explain this finding is that Asian-Americans have lower BMI compared with that of Whites, African-Americans and Hispanics/Latinos. In our study population, Asian-Americans did have lower BMI compared with other race/ethnic groups. However, even after re-categorising into ‘Asian-Americans’ and ‘not Asian-Americans’ (ie, Whites/African-Americans/Hispanics), Asian-Americans had a significantly lower prevalence of HH (PR=0.4 (95% CI 0.2 to 0.6)), after adjusting for age, sex and BMI indicating that lower BMI among Asian-Americans does not fully account for their lower prevalence of HH.

From a clinical standpoint, HH was strongly associated with proton pump inhibitor use, which is among the most common therapies for GERD.^3^ This supports HH detected on CT as an important structural cause of GERD. Interestingly, only one participant with HH at Exam 1 underwent surgical repair. This may reflect a substantial prevalence of asymptomatic or minimally symptomatic HH, the efficacy of medical management of GERD and also reticence to pursue surgical repair. Also of note, although alcohol use is an important risk factor for GERD,^1^ alcohol use showed no association with HH. In addition, HH was not associated with cigarette smoking status.

Strengths of our study include the highly reproducible quantitative and qualitative measures of HH on non-contrast full-lung and cardiac CT scans, and application within a large, highly characterised, multiethnic, US general population-based sample with 10-year follow-up. The major limitation of our study is the lack of a gold standard against which our HH observations could be validated. Similarities to prevalence measures from endoscopy-based studies are reassuring and probably all type III HH are accurately identified by CT. But CT may be insensitive to small, sliding HH as defined by surgery or high-resolution manometry.^36^ Assessment of Exam 1 cardiac CT scans was additionally limited by the lower resolution of multiplanar reformations, although type III and type IV HHs were readily identified on axial images and the prevalence results were similar between Exam 1 and the higher-resolution MESA Exam 5 CT. One advantage of CT was the high reproducibility of typing and quantifying HH size.

In conclusion, HH is detected on non-contrast CT with high reproducibility. It is prevalent in the general population, increasing with age, female gender and BMI similar to results from endoscopy studies of HH. Increasing incidence of HH with loss of height is consistent with the known association of HH with vertebral compression fractures. Association of detecting HH on CT with proton pump inhibitor use confirms a role in GERD and the association of CT-detected HH progression with BMI is important prognostically.
